# Baicalein from *Scutellaria baicalensis* mitigates oxidative stress through the IIS pathway in a *C. elegans* model of ulcerative colitis

**DOI:** 10.3389/fphar.2025.1592244

**Published:** 2025-06-26

**Authors:** Wei Wang, Xiaoyu Fu, Jianing Xu, Weiguang Lv, Shengnan Han, Yi Wang, Yu Xia, Jing Han, Ke Li, Chenggang Zhang

**Affiliations:** ^1^ School of Life Sciences, Beijing University of Chinese Medicine, Beijing, China; ^2^ Department of Orthopaedics, Chinese PLA General Hospital, Beijing, China

**Keywords:** *C. elegans*, ulcerative colitis, dextran sodium sulfate, baicalein, IIS pathway

## Abstract

**Introduction:**

Ulcerative colitis (UC) is a chronic, nonspecific inflammatory bowel disease with limited therapeutic options. Baicalein, a phenolic flavonoid extracted from *Scutellaria baicalensis*, has been traditionally used in Chinese medicine for its potent anti-inflammatory, anti-tumor, and antiviral properties. This plant, known as Huang-Qin, is indigenous to East Asia and has been widely used to treat various conditions such as fever, respiratory diseases, and inflammation.

**Aim of the Study:**

This study aimed to establish a *C. elegans* model of UC induced by dextran sodium sulfate (DSS) and to investigate the protective effects of baicalein on intestinal injury.

**Materials and Methods:**

DSS was used to induce acute intestinal injury in *C. elegans*. N2 and mutant strains (*daf-2* and *daf-16*) were exposed to DSS at concentrations of 5% (w/v), which identified as optimal for inducing intestinal inflammation. The effects of 25 μM, 50 μM, and 100 μM of baicalein on intestinal barrier function, oxidative stress markers, and relevant gene expression were evaluated, including genes related to epithelial barrier integrity (*clc-2*, *mtm-6*, etc.), oxidative stress, and the IIS and p38 MAPK pathways.

**Results:**

Baicalein significantly improved physiological condition and intestinal permeability in worm treated with 5% DSS. It restored the expression of epithelial barrier genes and reduced oxidative stress, as indicated by decreased ROS, enhancing SOD activity, daf-16 nuclear translocation etc. Baicalein’s protective effects were associated with the activation of the p38 MAPK and IIS pathways. In *daf-2* and *daf-16* mutant strains, baicalein demonstrated partial dependence on the IIS pathway for its protective effects.

**Conclusion:**

This study established a DSS-induced UC model in *C. elegans* and demonstrated that baicalein exerts protective effects on intestinal barrier integrity and oxidative stress, through the IIS and MAPK pathways. These findings support the use of *C. elegans* as a model for UC research and provide valuable insights into baicalein’s therapeutic potential for inflammatory bowel diseases.

## 1 Introduction

Ulcerative colitis (UC), a chronic inflammatory bowel disease (IBD), presents significant challenges in treatment due to its complex and persistent nature (Kornbluth et al., 2010). UC is characterized by typical clinical symptoms such as bloody diarrhea, and approximately one-third of UC patients experience extraintestinal manifestations. These can include hepatic complications, hematological disorders, and even colorectal cancer, contributing to reduced life expectancy (Kornbluth et al., 2010; Voelker, 2024). Over the past decades, a notable rise in UC incidence has been observed in emerging economies, particularly in Asia (da Silva et al., 2014; Burisch et al., 2013; Ng et al., 2017).

Current therapeutic strategies for UC patients range from aminosalicylic acid derivatives, glucocorticoids, immunomodulators, to biologics. However, these treatment methods come with side effects that may affect multiple organs, including the digestive, skeletal muscle, and cardiovascular systems (Burri et al., 2020; Loftus et al., 2004; Wise, 2016; Klotz et al., 2015). Notably, prolonged use of infliximab monotherapy can result in a “loss of response” over time, with unrelieved symptoms and the emergence of negative serum antibodies. Although combining immunomodulatory agents can alleviate this issue, it imposes a significant financial burden (Rutgeerts et al., 2005; Hoffmann et al., 2004). This situation has prompted medical researchers to explore alternative treatments that are both effective and safe for UC patients.

Two commonly used models for studying UC involve chemical agents like dextran sodium sulfate (DSS) or 2,4,6-trinitrobenzene sulfonic acid (TNBS) administered to mice via gavage, a process requiring an extended experimental timeline. Another approach involves inducing UC phenotypes through genetic knockouts such as *IL-2*, *Mdr1a*, or *TCR* in mice. However, using gene knockout mouse models is more expensive. *C. elegans*, a frequently used model organism in disease research, offers several advantages due to its intestinal structural and functional similarities to humans. It allows for a shorter model induction period and high biological reproducibility. At the 99th Dahlem Conference on Infection, Inflammation, and Chronic Inflammatory Disorders in 2010, *C. elegans* was proposed as a potential animal model for studying host immunity and microbial pathogenic mechanisms. This suggests that *C. elegans* could be a valuable tool for studying UC. To our knowledge, this is the first time that DSS has been used to induce inflammatory bowel disease in worms (Irazoqui and Ausubel, 2010).

Baicalein, a flavonoid and an active component of several traditional Chinese medicinal herbs, including *Scutellaria baicalensis* (Huang Qin in Chinese), has significant medicinal value according to its anti-inflammatory, anti-tumor, and antiviral properties. Baicalein’s antioxidant effects are believed to be central to its therapeutic action (Chen et al., 2006; Gupta et al., 2022). In traditional Chinese medicine (TCM), *Scutellaria baicalensis* is used for clearing heat and dampness, purging fire, and detoxifying, making it suitable for treating UC of the damp-heat type. As one of its primary active components, baicalein provides theoretical support for its clinical application in treating UC within TCM. Studies have demonstrated that baicalein can mitigate the severity of DSS-induced UC in mouse and rat models by reducing oxidative stress and enhancing intestinal epithelial barrier function (Li Y. et al., 2022; Liang et al., 2019; Liu C. et al., 2020). Furthermore, research by Li et al. showed that 10, 20 and 40 mg/kg baicalein significantly improved the survival rate of fruit flies with DSS-induced colitis, restored reduced intestinal length, and decreased intestinal permeability (Li et al., 2024) Additionally, baicalein has been demonstrated to have significant effects in treating diseases such as UC, Alzheimer’s disease (AD), and myocardial injury caused by oxidative stress (Jadhav and Kulkarni, 2023; Cui et al., 2015; Liu B. et al., 2020). Numerous studies have shown that UC’s pathogenesis and progression are closely associated with signaling pathways such as MAPK, Nrf2/HO-1, NF-κB, and PI3K-AKT. These pathways play critical roles in regulating oxidative stress, immune reactions, and intestinal homeostasis. Their activation or inhibition can significantly influence UC development and severity, making them important therapeutic targets. Understanding the complex regulatory mechanisms of these pathways may provide insights into novel UC treatments. In *C. elegans*, key pathways related to oxidative stress and pathogen defense include the p38 MAPK pathway and the insulin/IGF-1 signaling (IIS) pathway, which are evolutionarily conserved in both nematodes and mammals. This study aims to investigate the potential alterations in these pathways in a DSS-induced *C. elegans* model of UC (Nakagawa et al., 2016; Kumar et al., 2022).

Based on these findings, we aim to establish a UC model in *C. elegans* using DSS. Our objective is to investigate the effects of baicalein on intestinal barrier function, oxidative stress, and transcription factor expression. This study will explore the possible mechanisms underlying baicalein’s therapeutic efficacy for UC.

## 2 Materials and methods

### 2.1 Strain acquisition and cultivation of *C. elegans*

The wild-type N2, *daf-2*, and *daf-16* strains were obtained from the *Caenorhabditis* Genetics Center (CGC) at the University of Minnesota, United States. These strains were maintained on Nematode Growth Medium (NGM) seeded with *Escherichia coli* strain OP50 and incubated at an optimal temperature of 20°C.

### 2.2 Establishment and modulation of the ulcerative colitis model in *C. elegans*

The nematodes were synchronized and cultured at 20°C for 72 h to reach the L4 larval stage. Harvested larvae were washed in M9 buffer into EP tubes, followed by thorough rinsing, repeated more than four times, to ensure sterility.

For UC model induction, dextran sodium sulfate (DSS, molecular weight ∼5,000 Da; BioDee, Beijing, China) was added to K buffer. Nematodes were exposed to 500 μL of DSS solutions at concentrations of 1%, 2%, 5%, 10%, and 30% (w/v) respectively, while the control group received an equivalent volume of K buffer. All groups were maintained at 20°C for 24 h to allow UC symptoms to develop. Nematodes designated for intervention were then transferred onto fresh NGM plates containing 50 μM 5-fluorouracil (5-FU), either with or without baicalein (CAS 491-67-8; purity≥98%; Yuanye, Shanghai, China). These plates were incubated at 20°C for 48 h to assess baicalein’s protective effects. Baicalein was prepared at final concentrations of 25, 50, and 100 μM, dissolved in dimethyl sulfoxide (DMSO), with a standardized DMSO concentration of 0.1% (v/v) across all treatments.

### 2.3 Determination of the median lethal dose (LD50) of DSS on nematodes

*Caenorhabditis elegans* were cultured in 96-well plates in K solution containing varying concentrations of DSS: 0%, 1%, 2%, 5%, 10%, and 30%. The plates were incubated at 20°C for 24 h to assess DSS’s toxic effects. Nematodes were considered dead if they did not respond to gentle stimulation with a platinum wire. Each concentration group consisted of 120 nematodes, and the LD50 was calculated to determine the median lethal dose. This experiment was conducted in triplicate to ensure statistical reliability.

### 2.4 Assessment of body length and width

The body length and width of *C. elegans* were used as indicators of growth status (Han et al., 2022). After treatment, nematodes were transferred to microscope slides and anesthetized with 50 μL of a 10 mM levamisole solution. Dimensions were photographed and measured under somatoscopic microscopy (Chen et al., 2023). Head swing frequency, a behavioral indicator, was quantified by observing nematodes at 30-s intervals on M9 buffer-soaked slides. Each complete side-to-side head movement was recorded as a single event (Zhang JY. et al., 2024; Ye et al., 2021). Following head swing assessment, body bending frequency was recorded, defined as the number of bends per minute on an OP50-free NGM plate, tracking the posterior pharyngeal bulb along the y-axis (Han et al., 2022; Li et al., 2016; Zhang and Chen, 2023). 31 nematodes per group were evaluated, with each test repeated three times to ensure accuracy and reproducibility.

### 2.5 Lifespan assessment

The nematodes were maintained and treated as described above. To prevent interference from progeny, the treated nematodes were transferred to NGM plates supplemented with 50 μM 5-FU, a mitotic inhibitor (Macklin, Shanghai, China). This transfer marked the start of the experiment, denoted as day 0. For the first 5 days, nematodes were transferred daily to fresh 5-FU-NGM plates to prevent offspring proliferation, which could skew lifespan assessment. 37 nematodes per group were monitored daily. Death was determined by a lack of response to gentle prodding with a platinum wire and cessation of pharyngeal pumping. This experiment was conducted in triplicate to ensure reliability.

### 2.6 Assessment of intestinal permeability

Previous studies have shown that the epithelial cells of *C. elegans* share structural and functional similarities with mammalian cells, making them a suitable model for studying microbial dysbiosis, intestinal inflammation, and intestinal disease pathogenesis (Kim et al., 2022). Erioglaucine disodium salt (Sigma-Aldrich, United States), a recognized marker of intestinal barrier integrity (Qu et al., 2018; Gelino et al., 2016), was used to assess intestinal permeability. After 48 h of baicalein treatment, nematodes were washed three times with M9 buffer. They were then immersed in a 2.5% (w/v) staining solution at 20°C for 3 h. Subsequently, 50 μL of a 10 mM levamisole solution was added to microscope slides for observation under a super-resolution microscope (Leica, Wetzlar, Germany) with ×10 and ×40 objectives (Wu et al., 2023; Zhu et al., 2019).

Data were analyzed using the method by Ma Xuan et al., modified to account for potential erioglaucine leakage from the intestine into the body cavity due to intestinal injury (Ma, 2022). The erioglaucine blue damage coefficient was calculated as the ratio of the erioglaucine-stained area to total nematode area. The relative damage coefficient was determined by dividing the experimental group’s damage coefficient by that of the control group. The data, in TIFF format, were analyzed using ImageJ software (version 1.51; National Institutes of Health, United States) to calculate the percentage of positive blue signal area. Each group was measured in triplicate and 31 nematodes were examined per treatment group (Wu et al., 2023; Yu et al., 2020a).

### 2.7 Quantification of lipofuscin and reactive oxygen species (ROS) fluorescence

Lipofuscin, a pigment associated with aging, accumulates within nematodes over time (Yu et al., 2021; Qi et al., 2021). Nematodes were maintained and processed as previously described. After collection, they were washed with M9 buffer and incubated with 10 μM DCFH-DA (Nanjing Jiancheng Bioengineering Institute, China) probe for 2 h to assess ROS levels. Following incubation, nematodes were washed extensively to remove any residual probe and then placed on clean slides. For observation, 50 μL of a 10 mM levamisole solution was applied. ROS fluorescence was measured at an excitation wavelength of 488 nm and an emission wavelength of 525 nm.

To assess lipofuscin autofluorescence, nematodes treated with DSS or baicalein were similarly washed to minimize background fluorescence from bacterial debris. Fluorescence intensity was observed under blue excitation light (405–488 nm). 31 nematodes per group were evaluated and analyzed in triplicate using ImageJ software (Oh et al., 2022; Duangjan and Curran, 2022).

### 2.8 Assessment of superoxide dismutase (SOD) and malondialdehyde (MDA) levels

To evaluate oxidative stress, levels of SOD and MDA in the treated nematodes were measured. Approximately 1,000 nematodes were washed with M9 buffer, homogenized, and the supernatant was collected. Protein concentration was determined using the bicinchoninic acid (BCA) method. SOD and MDA levels were measured using commercial assay kits (Nanjing Jiancheng Bioengineering Institute, China) following the manufacturer’s protocols (Ji et al., 2022; Pei et al., 2022).

### 2.9 Nuclear localization assay of DAF-16

To assess the effects of low-, medium-, and high-dose baicalein on DAF-16GFP expression in *Caenorhabditis elegans* strain TJ356 exposed to 5% dextran sodium sulfate (DSS), treated worms were collected and washed repeatedly with M9 buffer, followed by immobilization on glass slides using 10 mM levamisole. GFP fluorescence was captured under consistent exposure settings (excitation wavelength: 485 nm, emission wavelength: 535 nm) using a fluorescence microscope. Fluorescence intensity was subsequently quantified using ImageJ software.

As previously described (Feng et al., 2021), the nuclear localization of DAF-16GFP was classified into three distinct patterns: cytosolic (diffuse cytoplasmic fluorescence), intermediate (partial nuclear accumulation), and nuclear (clear nuclear enrichment). Each treatment group included 20 worms, and experiments were performed in triplicate to ensure statistical rigor.

### 2.10 Quantitative real-time polymerase chain reaction (qRT-PCR)

To assess gene expression, qRT-PCR was conducted on the N2 strain and *daf-2* and *daf-16* mutant nematodes exposed to 5% DSS, with and without 50 μM baicalein treatment. Total RNA was extracted and purified using the RNA Easy Fast Tissue/Cell Kit (Tiangen, Beijing, China), following the manufacturer’s protocol. RNA purity and concentration were determined by measuring the 260/280 nm absorbance ratio, with acceptable values ranging from 1.8 to 2.2, using a NanoDrop™ spectrophotometer (Thermo Fisher Scientific, Waltham, MA, United States). cDNA synthesis was performed using the First-strand Synthesis Mast Mix kit (Lablead, Beijing, China). qRT-PCR was subsequently conducted using the 2× Realab Green PCR Fast mixture kit on a QuantStudio6 Flex system (Thermo, Beijing, China). Primers, with sequences and functions detailed in Supplementary Table S1, were synthesized by Sangon Biotech (Shanghai, China). Expression of the *act-1* gene was used as an internal control. A minimum of 1,000 *C. elegans* were used per group, and all experiments were performed in triplicate to ensure reproducibility (Fan et al., 2023; Dilberger et al., 2019).

### 2.11 Statistical analyses

The numerical data obtained were subjected to statistical analysis. Comparisons between two groups were conducted using Student’s t-test, while one-way ANOVA or non-parametric tests were applied as appropriate using SPSS version 27.0.1 (International Business Machines Corporation, American). In all analyses, statistical significance was defined at p < 0.05. Data visualization was performed using GraphPad Prism 8 (GraphPad Software, San Diego, CA, United States).

## 3 Results

### 3.1 Optimal modeling of ulcerative colitis in *C. elegans* with 5% DSS

**FIGURE 1:**
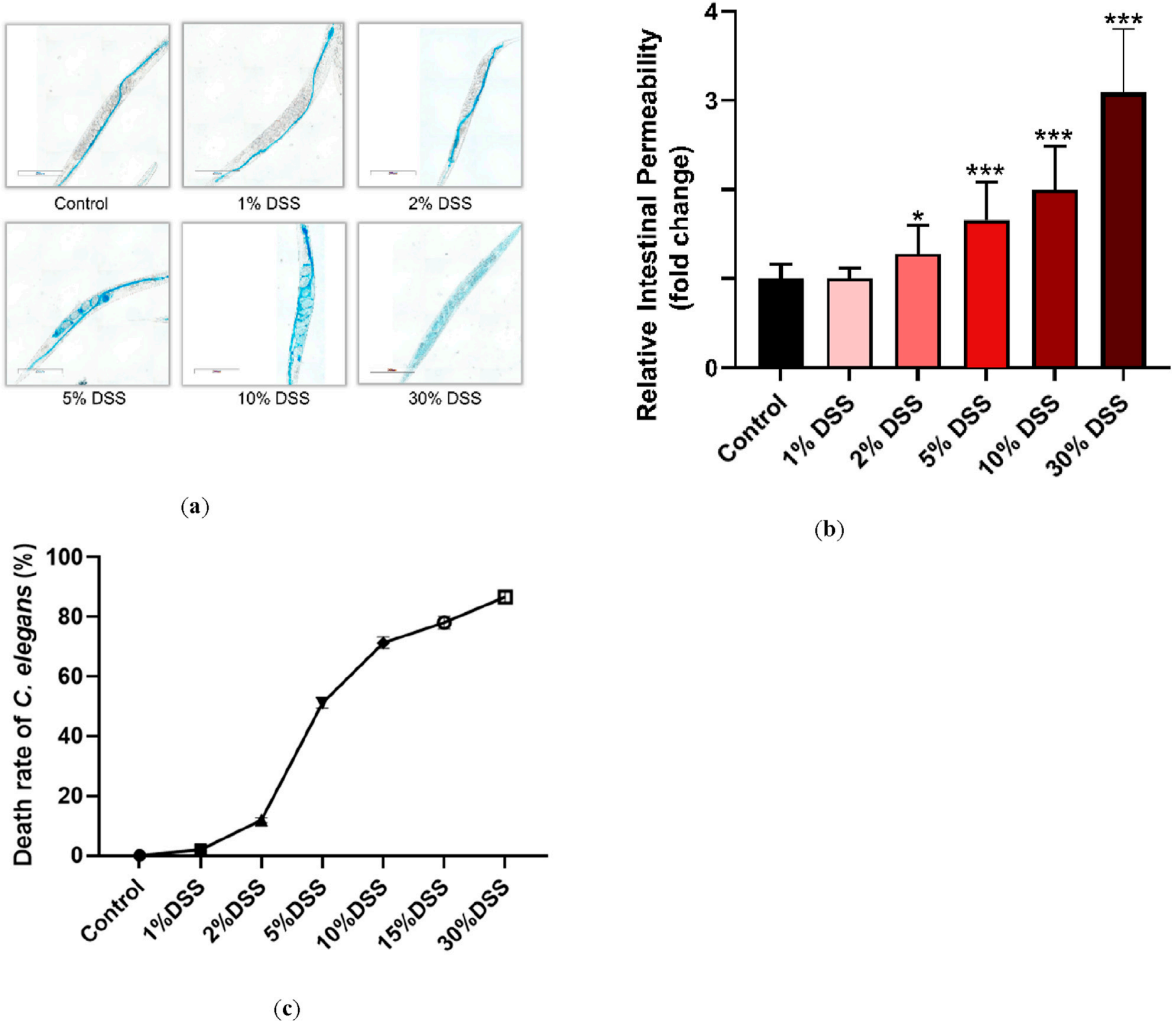
Effects of different DSS concentrations on ***C.*** *elegans*. Fourth-stage (L4) N2 *C. elegans* were exposed to 1%, 2%, 5%, 10%, and 30% DSS solutions for 24 h. Intestinal permeability was assessed following a rinse with M9 buffer (total magnification: ×100 and scale bar: 200 μm) **(a)**. **(b)**: Erioglaucine dye coverage on the nematodes was quantified to normalize the data. **(c)**: LD50 values for each DSS concentration on *C. elegans* were determined. Statistical significance is denoted by '*' for comparison to the control group, where *p < 0.05 and ***p < 0.001 indicate levels of significance.

Under normal conditions, the erioglaucine dye is selectively taken up by the intestine of *C. elegans*, showing minimal leakage outside the intestinal tract when the barrier is intact. However, a slight seepage of erioglaucine from the pharynx was observed in nematodes treated with 2% DSS compared to untreated controls. More pronounced leakage, indicated by larger areas stained with erioglaucine, was observed in nematodes exposed to 5%, 10%, and 30% DSS concentrations, with significant differences in relative damage coefficients (Figures 1a,b). Notably, DSS concentrations of 10% and higher resulted in a mortality rate over 74%.

To balance experimental feasibility with concentrations commonly reported in the literature, the 5% DSS concentration was selected for further studies. This concentration corresponds to the half-lethal dose (LD50) and effectively induces intestinal barrier damage, making it a suitable choice for modeling UC in *C. elegans* (Figure 1c) (Prakash and Janadri, 2023; Low et al., 2013; Kim et al., 2023; Munyaka et al., 2016).

### 3.2 Baicalein ameliorates the growth and development of DSS-Treated *C. elegans*

**FIGURE 2:**
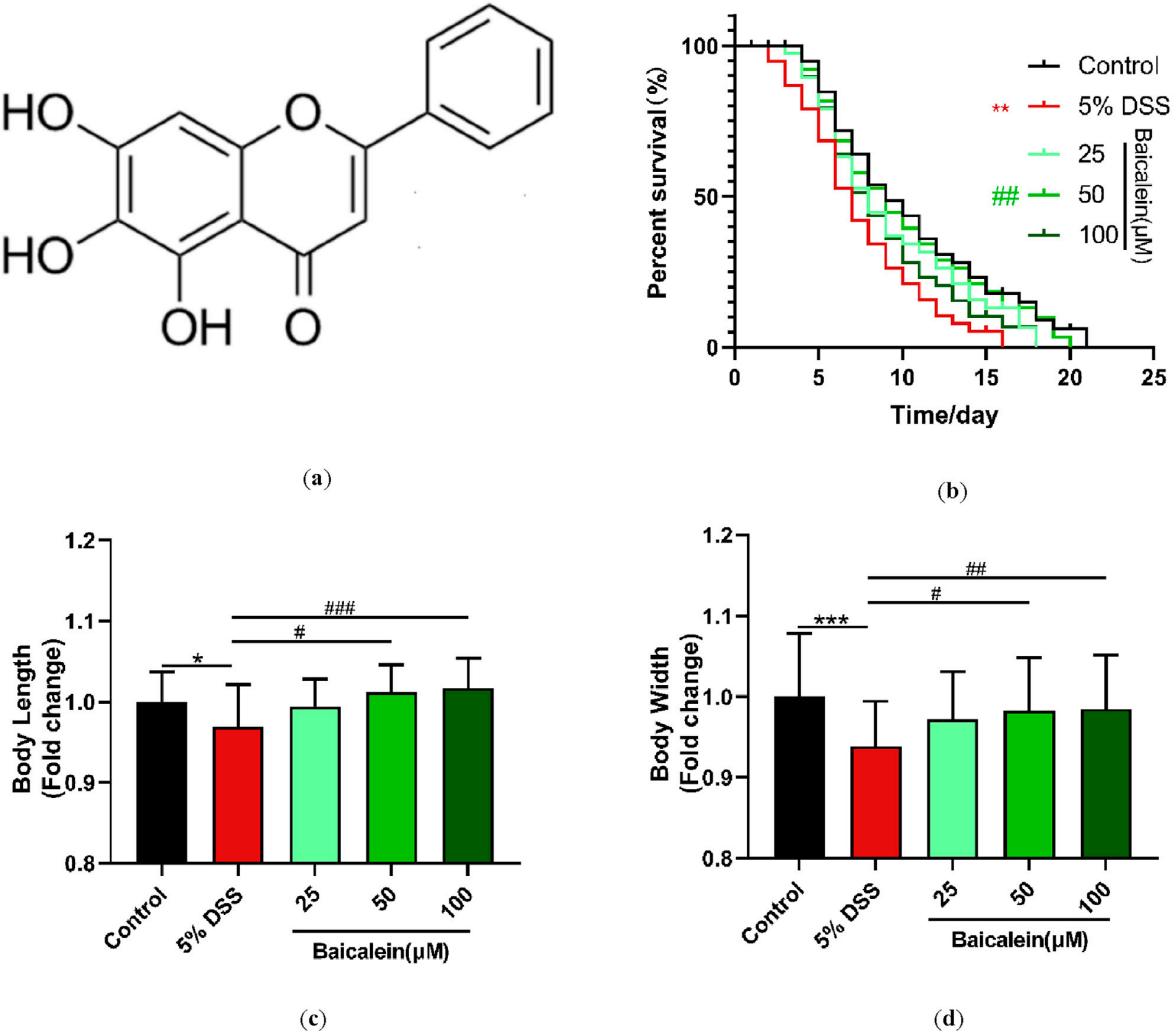
Baicalein intervention promotes growth and development in nematodes following DSS treatment. **(a)** Molecular structure of baicalein. L4 stage N2 *C. elegans* were treated with 5% DSS followed by 0.1% DMSO or varying concentrations of baicalein (25, 50, and 100 μM). Longevity **(b)**, body length **(c)**, and body width **(d)** were assessed, with data normalized accordingly. Statistical significance is denoted by '*' for comparisons with the control group and '#' for comparisons with the 5% DSS treatment group, with *p < 0.05, ***p < 0.001, #p < 0.05, ##p < 0.01, and ###p < 0.001 indicating significance levels.

The beneficial physiological impact of baicalein (Figure 2a) on DSS-exposed nematodes was evaluated using three metrics: lifespan, body length, and body width. Our findings showed that treatment with 5% DSS markedly decreased the average lifespan of N2 nematodes by 27.3% compared to control group, reflecting a stressed physiological state (Figure 2b). However, a 25.1% increase in lifespan was observed after the 48-h treatment with 50 μM baicalein (Supplementary Table S2).

Regarding body dimensions, the mean body length and width of the control nematodes were 657.85 ± 2.46 μm and 47.01 ± 0.37 μm, respectively, while the 5% DSS-treated group measured 637.48 ± 3.44 μm in length and 44.14 ± 0.26 μm in width (Supplementary Table S3). Intervention with both 50 μM and 100 μM baicalein significantly counteracted these detrimental effects, with no significant difference observed between the two concentrations (p > 0.05) (Figures 2c,d).

These results indicate that 5% DSS exposure negatively affects the growth and developmental parameters of *C. elegans*. Furthermore, moderate to high concentrations of baicalein (50 and 100 μM) demonstrate a significant restorative effect on growth impairment of *C. elegans* induced by DSS. Previous studies have indicated that *C. elegans* models are reliable for testing UC therapeutic agents, particularly given their conserved genetic pathways related to stress and immunity, which parallel mammalian systems.

### 3.3 Baicalein enhances locomotion in DSS-Treated *C. elegans*

**FIGURE 3:**
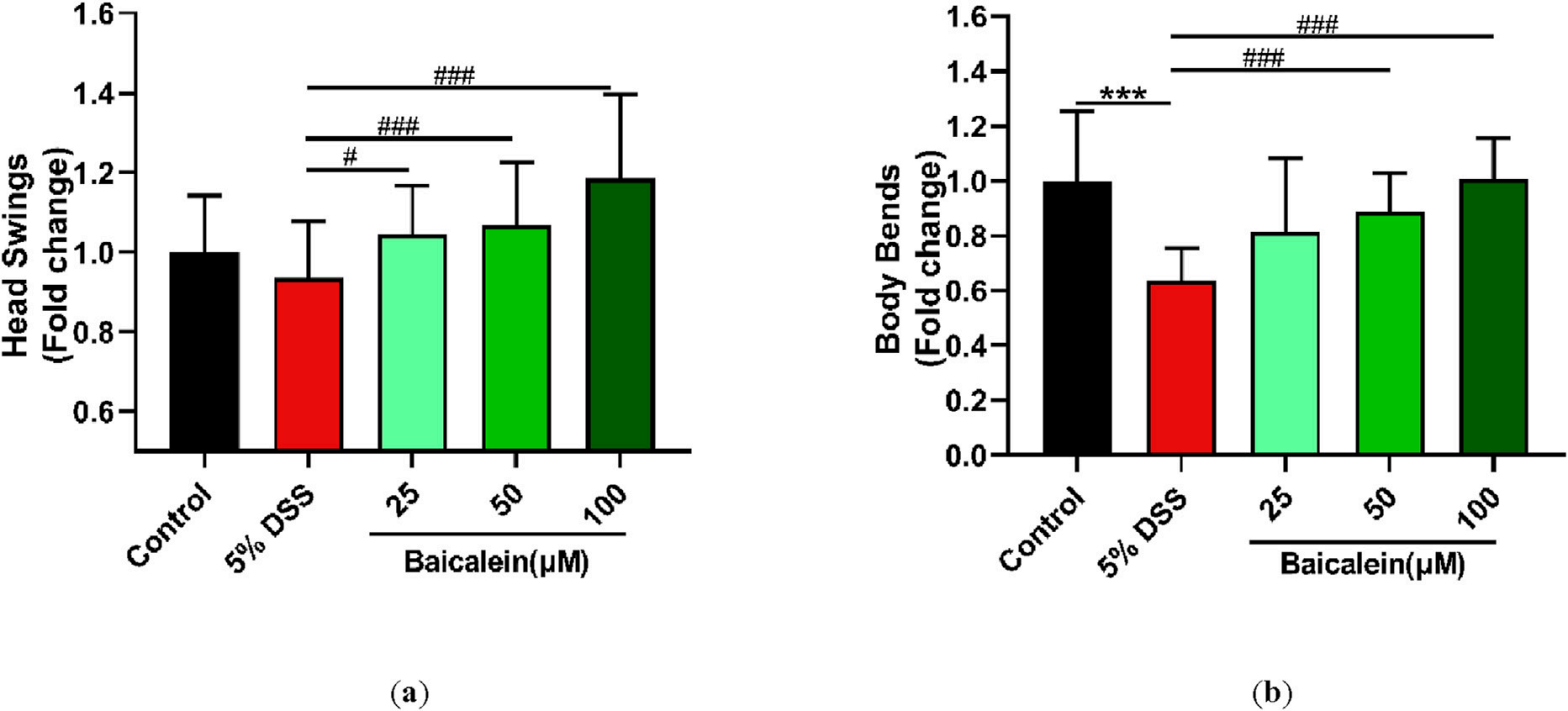
Baicalein ameliorates movement impairments in nematodes following DSS treatment. L4 N2 *C. elegans* exposed to 5% DSS were treated with 0.1% DMSO or baicalein (25, 50, and 100 μM) respectively. Locomotive abilities were evaluated by measuring head thrashes per 30 s **(a)** and body bends per minute **(b)**, with normalized data for comparison. Statistical significance is denoted by '*' for comparisons with the control group and '#' for comparisons with the 5% DSS treatment group, with *p < 0.05, ***p < 0.001, #p < 0.05, ##p < 0.01, and ###p < 0.001 indicating levels of significance.

Assessment of movement behavior, including head thrashing and body bending, is critical for evaluating the locomotive capacity of *C. elegans*, as these locomotion behavior in *C. elegans* is often used as a measure of neuromuscular function and overall vitality (Liu H. et al., 2022). Our results demonstrated that 50 and 100 μM baicalein significantly improved impaired movement behaviors impaired by 5% DSS exposure, specifically increasing the frequency of body bends per minute and head thrashes per 30 s (p < 0.001). Interestingly, a 25 μM concentration of baicalein improved head thrashing (p < 0.05) without significantly affecting body bending (p > 0.05) (Figures 3a,b). These findings indicate that baicalein effectively enhances the physiological state of N2 nematodes following DSS-induced stress.

### 3.4 Baicalein reverses intestinal permeability and gene expression in DSS-Treated nematodes

**FIGURE 4:**
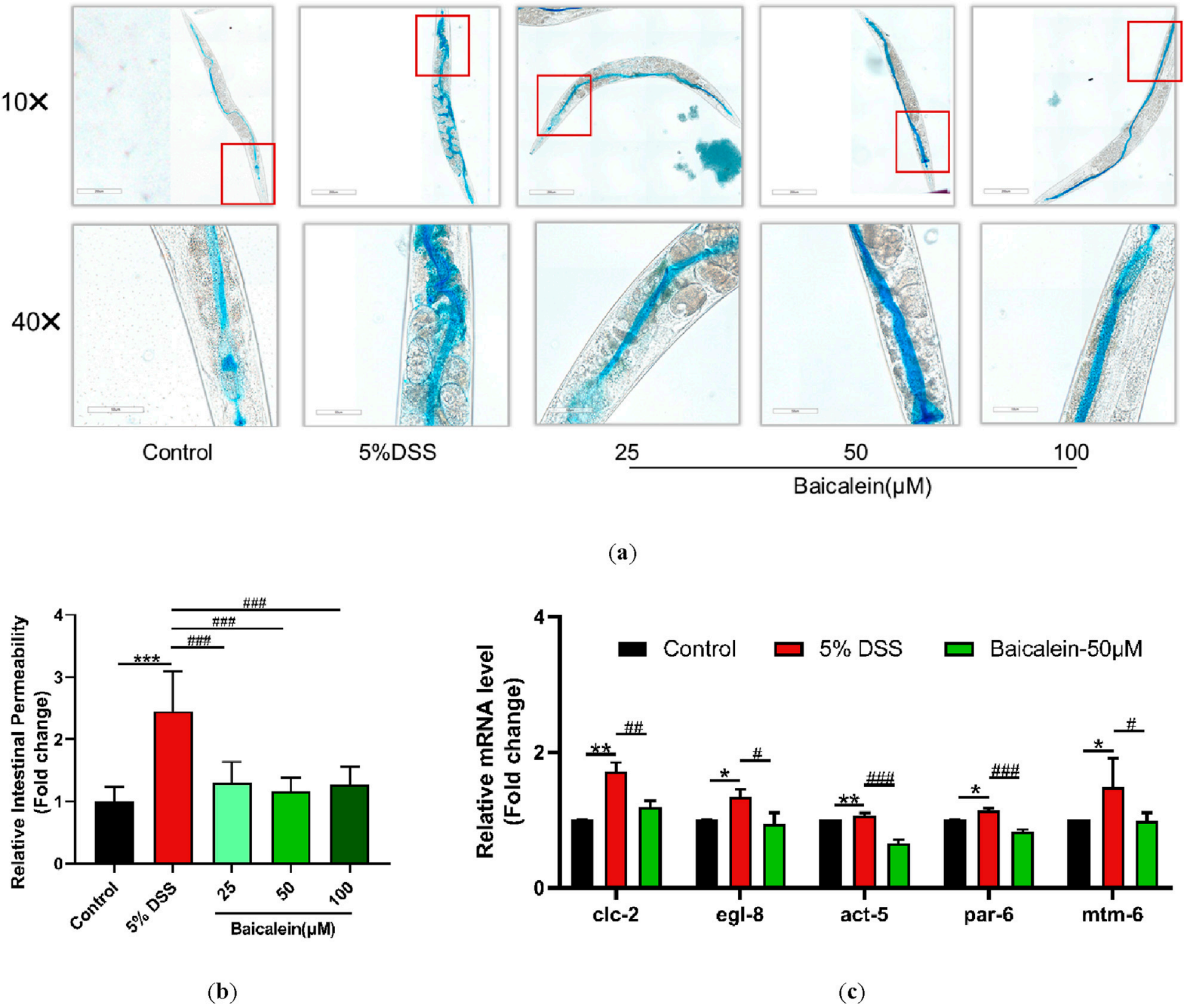
Baicalein mitigates intestinal permeability in DSS-treated ***C.*** *elegans*. The effects of baicalein treatment on *C. elegans* exposed to 5% DSS were assessed by observing L4 stage nematodes treated with either 0.1% DMSO or varying concentrations of baicalein (25, 50, and 100 μM). **(a)** Intestinal permeability was observed with total magnification ×100 (scale bar: 200 μm) and ×400 (scale bar: 50 μm) magnifications. Quantification of the observed effects is shown in **(b)**. **(c)** qRT-PCR analysis was used to assess expression levels of genes involved in intestinal barrier integrity, with data normalized for comparison. Statistical significance is indicated by '*' for comparisons with the control group and '#' for comparisons with the 5% DSS treatment group, with notations denoting significance levels: *p < 0.05, **p < 0.01, ***p < 0.001, #p < 0.05, ##p < 0.01, ###p < 0.001.

Maintaining intestinal barrier integrity is a crucial factor in UC pathology. In our study, 5% DSS treatment led to a significant infiltration of erioglaucine dye throughout the bodies of N2 nematodes, indicating a substantial increase in intestinal permeability. In contrast, treatment with various concentrations of baicalein resulted in no noticeable dye accumulation outside the intestine (Figures 4a,b). Moreover, qRT-PCR analysis revealed that treatment with 50 μM baicalein effectively restored expression levels of genes associated with intestinal barrier function, such as *clc-2*, *egl-8*, *act-5*, *par-6*, and *mtm-6*, which had been upregulated following DSS exposure (Figure 4c). Collectively, these findings demonstrate that while DSS treatment severely disrupts intestinal barrier function in N2 nematodes, subsequent baicalein intervention significantly alleviates this damage.

### 3.5 Baicalein reduces lipofuscin and ROS accumulation in DSS-Treated nematodes

**FIGURE 5:**
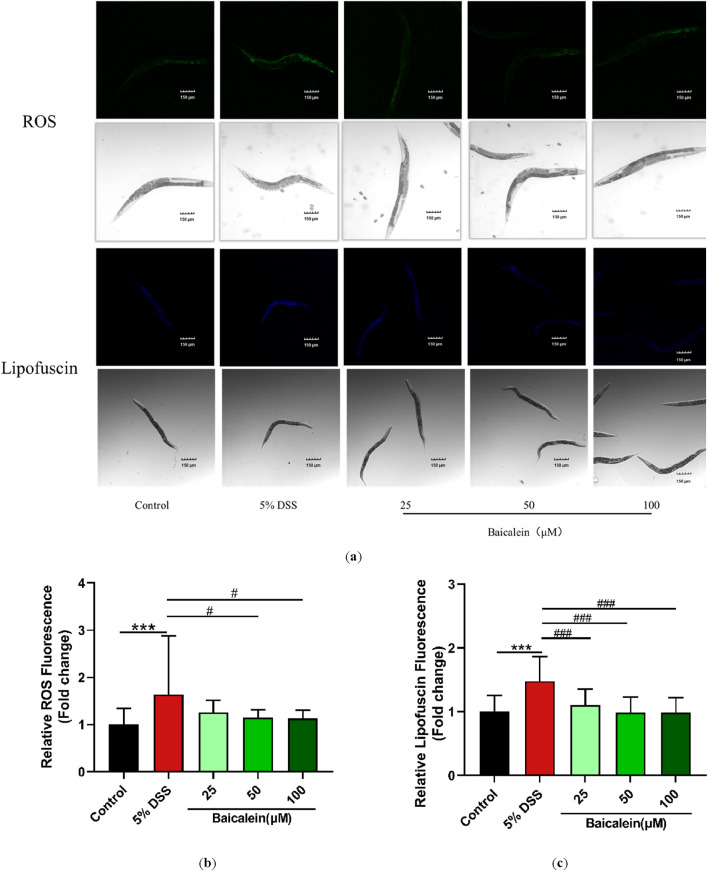
Baicalein reduces oxidative stress in DSS-exposed *C. elegans*. The L4 stage of N2 *C. elegans* exposed to 5% DSS were treated with either 0.1% DMSO or baicalein (25, 50, and 100 μm) to assess oxidative stress. Lipofuscin and ROS levels were observed under fluorescence microscopy with total magnification ×100 (scale bar: 150 μm) **(a)**. Fluorescence intensities were quantified for ROS **(b)** and lipofuscin **(c)** with data normalized for comparison. Statistical significance is denoted by '*' for comparisons with the control group and '#' for comparisons with the 5% DSS treatment group, with significance indicated as ***p < 0.001, #p < 0.05, ###p < 0.001.

Fluorescence microscopy with ImageJ quantification revealed that exposure to 5% DSS increased lipofuscin fluorescence intensity by 48% compared to untreated controls, an effect that was significantly counteracted by baicalein treatment at concentrations of 25, 50, and 100 μM (Figures 5a,c). Additionally, we assessed baicalein’s impact on ROS production in DSS-treated nematodes. Fluorescence analysis of approximately 30 nematodes per group revealed intensified green fluorescence primarily around the intestines of nematodes treated with DSS alone. DSS exposure resulted in a 64.42% increase in ROS levels, which baicalein intervention reduced by 29.77% and 31.19% at concentrations of 50 and 100 μM, respectively (Figure 5b).

### 3.6 Baicalein modulates MDA and SOD levels in DSS-Treated nematodes

**FIGURE 6:**
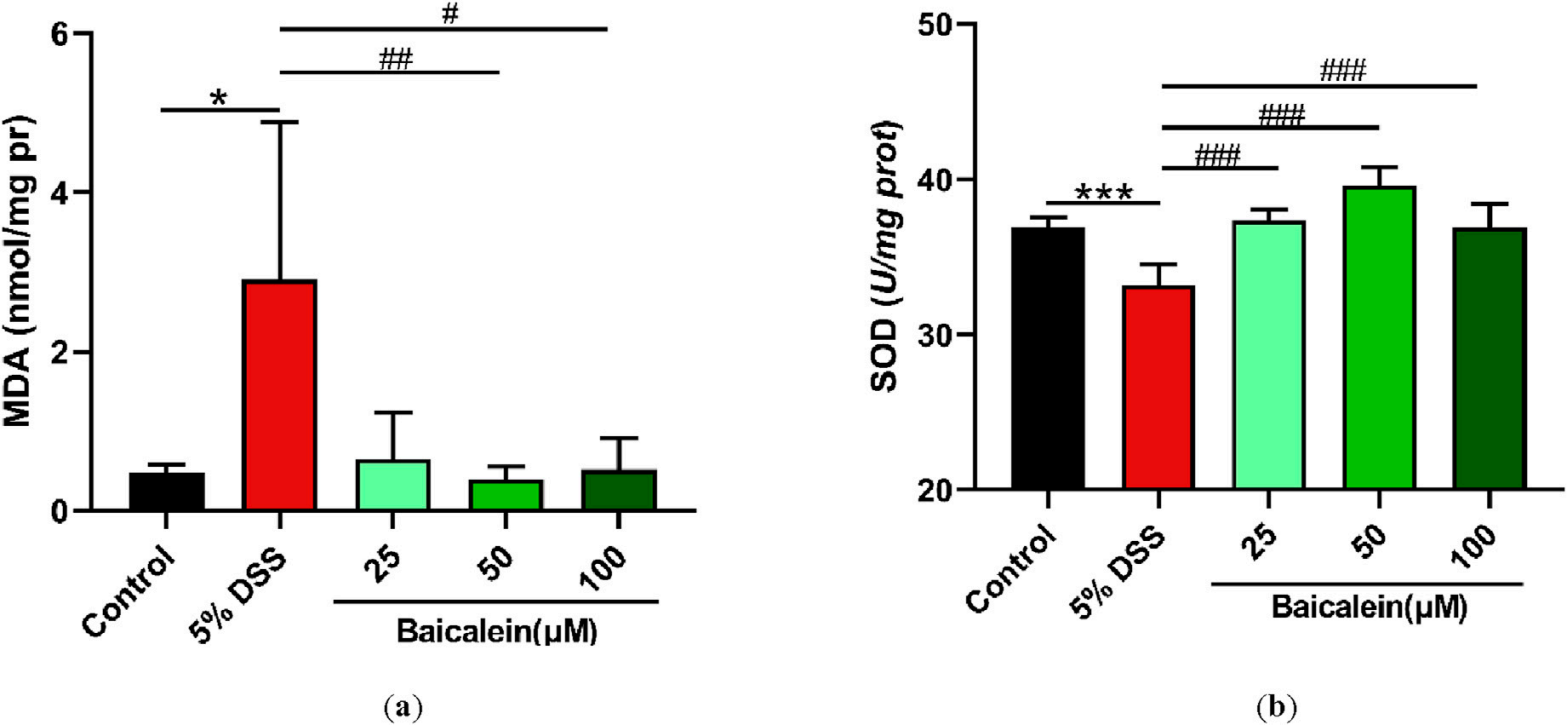
Baicalein alleviates oxidative stress in DSS-treated *C. elegans* by modulating oxidative and antioxidant markers. The L4 stage of N2 *C. elegans* exposed to 5% DSS were treated with 0.1% DMSO or baicalein (25, 50, and 100 μM). Levels of MDA **(a)** and SOD **(b)** were measured. Statistical significance is denoted by '*' for comparisons with the control group and '#' for 5% DSS treatment group comparisons, with notations indicating significance: *p < 0.05, ***p < 0.001, #p < 0.05, ##p < 0.01, ###p < 0.001.

Further investigations focused on measuring MDA and SOD levels in cell lysates from 5% DSS treated nematodes. MDA, a marker of lipid peroxidation, is an indicator of oxidative stress (Liu Y. et al., 2022; Xiao et al., 2021; Zhang X. et al., 2024). Exposure to 5% DSS resulted in a notable 6-fold increase in MDA levels compared to the control group incubated in K solution for 24 h (p < 0.05). Although low-dose of baicalein (25 μM) exhibited a tendency to reduce MDA, more pronounced reductions were observed with medium and high doses of baicalein (50 and 100 μM, respectively) (p < 0.05) (Figure 6a).

Additionally, we evaluated the changes in SOD activity, a critical antioxidant enzyme. The 5% DSS treatment caused a significant 10.14% decrease in SOD activity in N2 nematodes, whereas baicalein treatment at 25, 50, and 100 μM increased SOD activity by 12.5%, 19.45%, and 11.14%, respectively (Figure 6b). Although baicalein at 50 μM exhibited the highest SOD activity restoration, the difference between 50 μM and 100 μM groups did not reach statistical significance (p > 0.05), suggesting a plateau or concentration-dependent divergence in its regulatory effects on SOD.

### 3.7 Baicalein modulates p38 MAPK pathway gene expression in DSS-Treated nematodes

**FIGURE 7:**
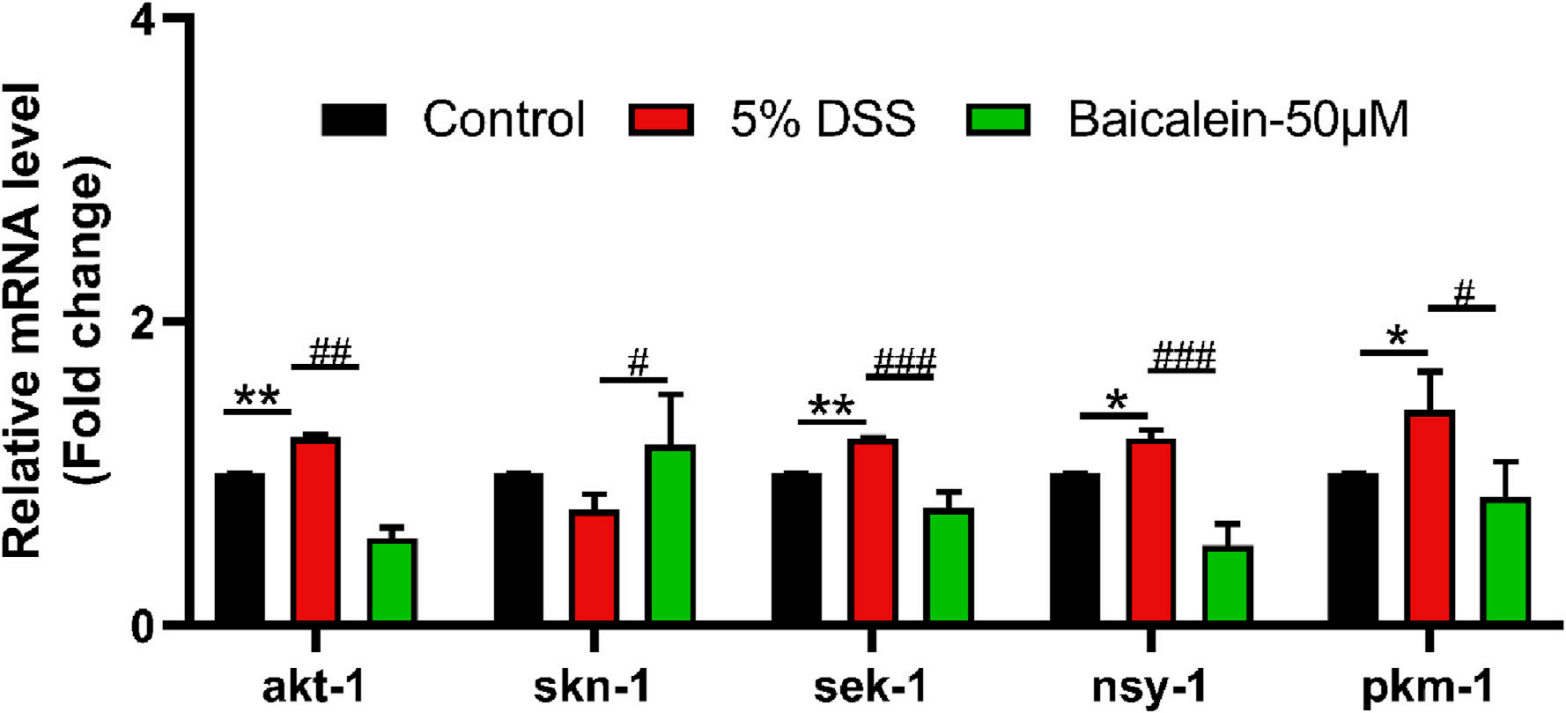
Baicalein modulates gene expression within the p38 MAPK pathway in DSS-exposed *C. elegans*. The L4 stage of N2 *C. elegans* subjected to 5% DSS were treated with 0.1% DMSO or baicalein (50 μM). mRNA levels of p38 MAPK pathway genes were measured in nematode homogenates. Statistical significance is indicated by '*' for comparisons with the control group and '#' for DSS model group comparisons, with significance denoted as *p < 0.05, **p < 0.01, #p < 0.05, ##p < 0.01, ###p < 0.001.

The MAPK pathway, crucial in UC progression, was assessed in the DSS-induced UC model. Results showed that 5% DSS treatment upregulated genes within the p38-MAPK pathway, including *sek-1* (MAPKK), *nsy-1* (MAPKKK), and *pmk-1* (MAPK) (Figure 7). Activation of this pathway typically increases expression of *Nrf-2/Skn-1*, which DSS did not alter significantly, though baicalein increased it at the concentration of 50 μM. Additionally, *akt-1* expression decreased by 53.20% following 50 μM baicalein treatment. These data support baicalein’s role in ameliorating DSS-induced intestinal barrier damage and oxidative stress, especially at 50 μM.

### 3.8 Baicalein reverses oxidative stress-related gene expression in DSS-Treated nematodes

**FIGURE 8:**
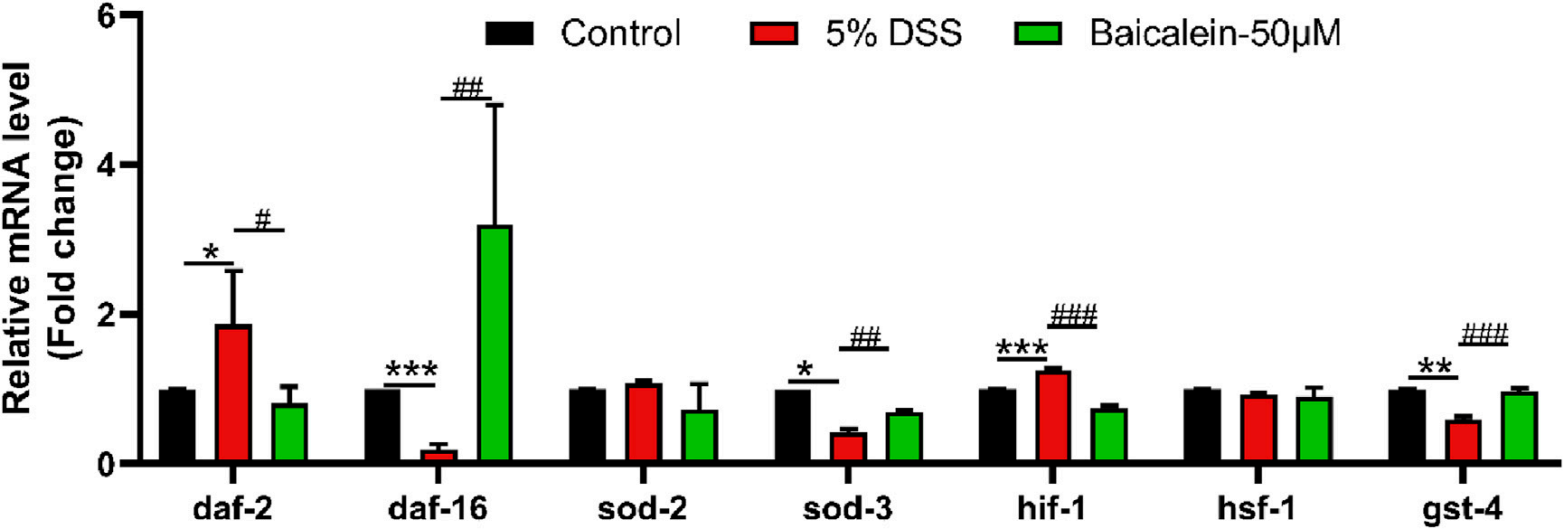
Baicalein alleviates oxidative stress in DSS-treated *C. elegans* by modulating oxidative stress-related mRNA. The L4 stage of N2 *C. elegans* exposed to 5% DSS were treated with 0.1% DMSO or baicalein 50 μM). Levels of oxidative stress-related mRNA was measured. Statistical significance is denoted by '*' for comparisons with the control group and '#' for 5% DSS treatment group comparisons, with notations indicating significance: *p < 0.05, **p < 0.01, ***p < 0.001, #p < 0.05, ##p < 0.01, ###p < 0.001.

The IIS pathway is a critical regulator of oxidative stress in *C. elegans* (Li H. et al., 2021; Zaarur et al., 2019). Oxidative stress plays a central role in UC pathogenesis (Elmaksoud et al., 2021; Nikkhah-Bodaghi et al., 2019; Elkholy et al., 2023). Our results (Figure 8) demonstrated that 5% DSS treatment significantly upregulated *daf-2* gene expression (analogous to mammalian insulin/IGF-1), while concurrently downregulating its downstream target *DAF-16/FOXO*, a forkhead box O transcription factor. This effect was reversed by 50 μM baicalein treatment. Furthermore, mRNA levels of antioxidant stress-related genes *sod-3* and *gst-4* (downstream of *DAF-16/FOXO*) were reduced by 58.02% and 40.52%, respectively, following DSS exposure, with subsequent increases of 63.03% and 67.91%, respectively, after intervention with 50 μM baicalein. In addition, baicalein reduced the DSS-induced upregulation of *hif-1* mRNA, though no significant changes were observed in *sod-2* and *hsf-1* gene expression.

### 3.9 Baicalein restores the nuclear translocation of DSS-Treated nematodes

**FIGURE 9:**
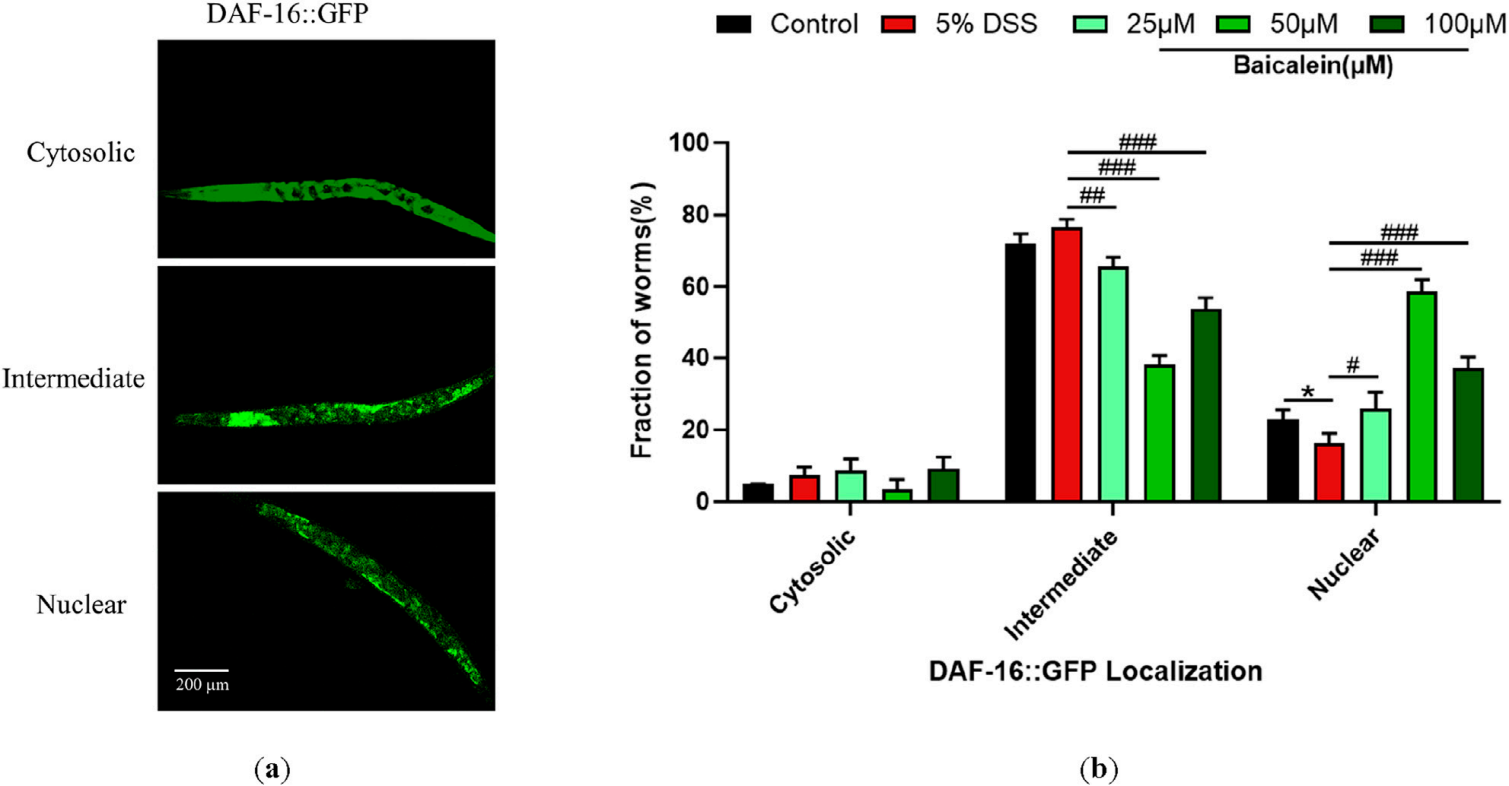
Baicalein influences subcellular localization of DAF-16::GFP. The L4 stage of the DAF-16GFP strains exposed to 5% DSS were treated with 0.1% DMSO or baicalein (25, 50, and 100 μM). **(a)** Fluorescence microscopy images depicting DAF-16GFP nuclear translocation events in TJ356 strain nematodes with total magnification ×100 (scale bar: 200 μm) **(b)** Quantitative analysis of nuclear translocation frequency for DAF-16GFP fusion protein. Statistical significance is indicated by '*' for comparisons with the control group and '#' for comparisons with the 5% DSS treatment group, with notations denoting significance levels: *p < 0.05, #p < 0.05, ##p < 0.01, ###p < 0.001.

Compared to the control group, the nuclear translocation of DAF-16 in TJ356 nematodes after 5% DSS exposure was significantly reduced by 29.38% (Figures 9a,b). The addition of baicalein significantly restored the nuclear localization of DAF-16 under DSS stress, especially at 50 μM.

### 3.10 Baicalein’s mechanism in DSS-Treated nematodes involves IIS pathway modulation

**FIGURE 10:**
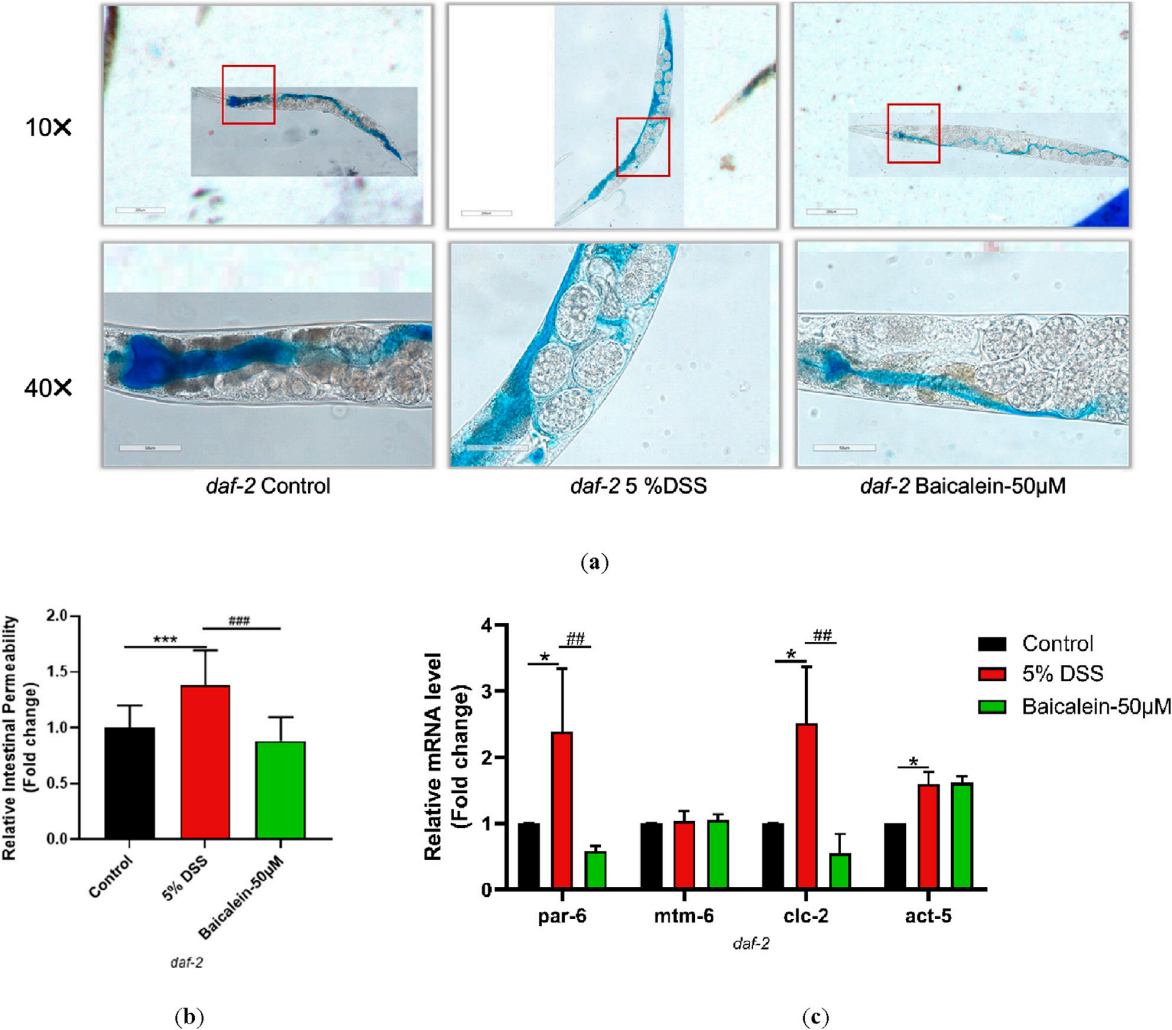
Baicalein Enhances Intestinal Barrier in DSS-Treated *daf-2* Mutant. The L4 stage of the *daf-2* mutant strains exposed to 5% DSS were treated with 0.1% DMSO or baicalein (50 μM). **(a)** Intestinal permeability was observed with total magnification ×100 (scale bar: 200 μm) and ×400 (scale bar: 50 μm) magnifications. Quantification of the observed effects is shown in **(b)**. **(c)** qRT-PCR analysis was used to assess expression levels of genes involved in intestinal barrier integrity, with data normalized for comparison. Statistical significance is indicated by '*' for comparisons with the control group and '#' for comparisons with the 5% DSS treatment group, with notations denoting significance levels: *p < 0.05, **p < 0.01, ***p < 0.001, #p < 0.05, ##p < 0.01, ###p < 0.001.

**FIGURE 11:**
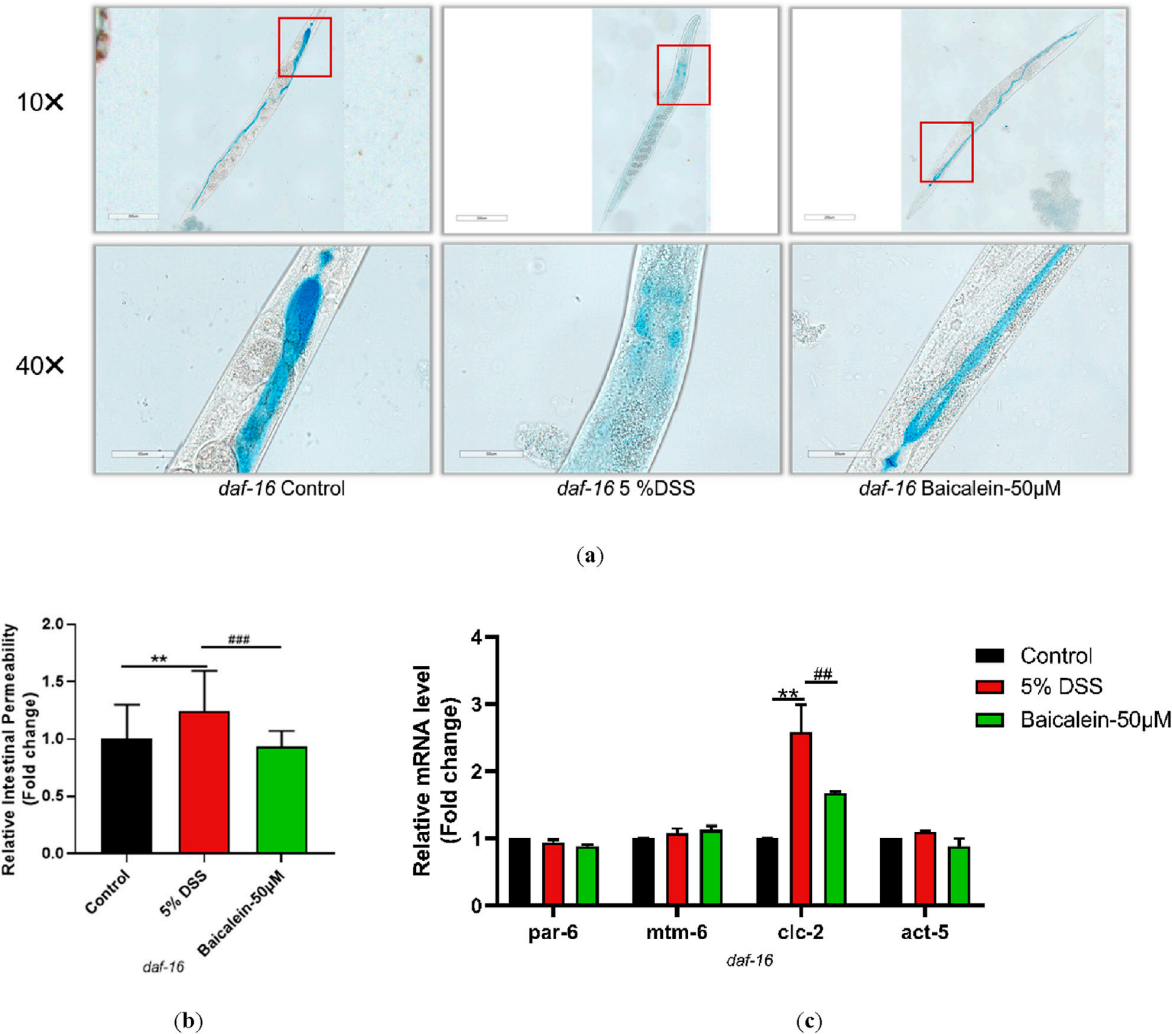
Baicalein Enhances Intestinal Barrier in DSS-Treated *daf-16* Mutant Nematodes. The L4 stage of the *daf-16* mutant strains exposed to 5% DSS were treated with 0.1% DMSO or baicalein (50 μM). **(a)** Intestinal permeability was observed with total magnification ×100 (scale bar: 200 μm) and ×400 (scale bar: 50 μm) magnifications. Quantification of the observed effects is shown in **(b)**. **(c)** qRT-PCR analysis was used to assess expression levels of genes involved in intestinal barrier integrity, with data normalized for comparison. Statistical significance is indicated by '*' for comparisons with the control group and '#' for comparisons with the 5% DSS treatment group, with notations denoting significance levels: *p < 0.05, **p < 0.01, ***p < 0.001, #p < 0.05, ##p < 0.01, ###p < 0.001.

To elucidate the mechanism underlying *daf-2* and *daf-16* gene induction and intestinal barrier response following baicalein treatment in DSS-exposed *C. elegans*, intestinal permeability and gene expression were examined in mutant strains of nematodes. Treatment of *daf-2* mutants with 5% DSS increased intestinal permeability and the expression of *par-6*, *clc-2*, and *act-5*, though *mtm-6* remained unchanged. Baicalein (50 μM) also did not alter *mtm-6* or *act-5* expression (Figure 10). In *daf-16* mutants, both DSS and baicalein treatments produced comparable effects on permeability, with no significant changes in *par-6*, *mtm-6*, or *act-5* mRNA levels (Figure 11).

## 4 Discussion

UC, with a projected global prevalence of approximately 5 million cases by 2023, poses significant challenges to both physical and mental health (Le Berre et al., 2023). Despite ongoing research, the precise etiology of UC remains elusive, with multiple factors including genetic predisposition, environmental triggers, immune dysregulation, disruptions to the intestinal barrier, and alterations in the gut microbiome being implicated (Wangchuk et al., 2024; Saez et al., 2023). One of the key pathological features of UC is damage to the intestinal epithelial barrier, often occurring during early disease flares. The current study underscores the potential of baicalein in mitigating UC symptoms using a *C. elegans* model induced by 5% DSS. The DSS model successfully mimics critical aspects of UC pathology, such as impaired barrier function and oxidative stress, thus providing a valuable platform for investigating baicalein’s therapeutic mechanisms.

Our findings suggest that baicalein exerts its protective effects through multiple pathways, particularly the IIS (insulin/IGF-1) and MAPK (mitogen-activated protein kinase) pathways. These pathways are crucial for regulating stress responses, immune function, and maintaining intestinal homeostasis. In *C. elegans*, the concentrations used in this study were chosen to approximate these ranges, taking into account the differences in body size, metabolism, and uptake mechanisms between nematodes and higher organisms. This approach ensures that the concentrations tested are biologically relevant and comparable to those shown to exert therapeutic effects in other models. Moreover, baicalein demonstrated a favorable safety profile, as evidenced by the normal lifespan, body dimensions, and movement behaviors of *C. elegans* treated with 5% DSS. Previous studies have also supported the safety of baicalein in various pharmacological contexts, Susannah Havermann et al., Saswat Kumar Mohanty et al. have demonstrated that intervention with 25 μM, 50 μM, and 100 μM baicalin significantly enhances the lifespan and stress resistance of *C. elegans* (Havermann et al., 2016).

Importantly, baicalein was observed to enhance intestinal barrier integrity, as indicated by reduced intestinal permeability and the upregulation of genes critical for barrier function. Increased intestinal permeability is a well-recognized hallmark of IBD, with studies indicating that patients with elevated permeability are more prone to disease relapse (Arnott et al., 2000). In the DSS-induced colitis model, mucosal permeability was found to increase prior to the onset of inflammation, underscoring the pivotal role of barrier dysfunction in UC progression (Kitajima et al., 1999). The methylene erioglaucine-stained *C. elegans* model employed in this study offered a more accessible and comprehensive method for evaluating intestinal barrier function across the full intestinal tract, as compared to traditional permeability assays in rodent models.

Baicalein’s ability to restore key intestinal barrier function genes, such as *clc-2* and *egl-8*, further supports its role in strengthening intestinal integrity. The *clc-2* gene, which shares homology with the claudin family in humans, plays a crucial role in cellular adhesion and signaling (Simske and Hardin, 2011), while *egl-8* is associated with the activation of the PMK-1/p38 MAPK pathway, which is integral to intestinal immunity (Yang et al., 2024; Miller et al., 1999). Notably, baicalein treatment resulted in decreased *egl-8* and increased *daf-16* expression, consistent with its role in modulating insulin-like signaling pathways and oxidative stress response (Mack et al., 2022). Furthermore, genes like *act-5*, which maintains the structural stability of microvilli, and *par-6*, involved in cell polarity, were transiently upregulated in response to DSS exposure, contributing to the observed barrier protection (Cheng et al., 2023; MacQuee et al., 2005; Yamanaka et al., 2001; Totong et al., 2007). Also, our study observed transient upregulation of *mtm-6* and *act-5* following DSS exposure, which contributes to barrier function enhancement and protection against toxicants (Yu et al., 2020b; Ren et al., 2018; Zhou et al., 2024; Wu et al., 2024). While baicalein’s restoration of intestinal permeability in daf-2 mutants—without altering mtm-6 expression—reveals additional complexity. In WT nematodes, mtm-6 induction likely depends on DAF-16 activation downstream of IIS, aligning with its role in membrane trafficking (Liu Y. et al., 2022). However, in daf-2 mutants, baicalein rescued permeability despite static mtm-6 mRNA levels, suggesting compensatory mechanisms such as: post-translational activation of MTM-6’s lipid phosphatase activity, which restores phosphatidylinositol-3-phosphate (PI3P) metabolic homeostasis. This promotes Wnt secretion, and ameliorates intestinal permeability (Li W. J. et al., 2021; Liu P. et al., 2020; Silhankova et al., 2010); or (2) DAF-16-independent pathways (PI3K/AKT) reinforcing junctions via par-6/clc-2. For instance, AKT-mediated phosphorylation of PAR-6 or its binding partners (e.g., CDC-42) could stabilize apical junctions independently of DAF-16. It may regulate CDC-42 GTPase activity, promoting PAR-6/PKC-3 complex assembly for maintaining junctional continuity during cell division and elongation (Sallee et al., 2021; Wymann and Pirola, 1998). This phenotypic-genetic decoupling highlights baicalein’s capacity to engage parallel networks—spanning IIS-dependent transcription and IIS-independent post-transcriptional regulation—to restore homeostasis. Such multi-target efficacy emphasizes the therapeutic advantage of flavonoids in complex diseases like UC.

When the intestinal barrier is impaired, neutrophils clear pathogens through phagocytosis, degranulation, ROS production, and neutrophil extracellular traps (NETs) release. However, cytokines and ROS released can disrupt intestinal epithelial tight junctions, activating pathways like p38-MAPK (Elkholy et al., 2023; Althagafy et al., 2023), PI3K/AKT (Zhang J. et al., 2022; Ma et al., 2023) and NF-κB, which in turn amplify oxidative stress and recruit more immune cells to inflamed tissue (McCracken and Allen, 2014; Nakase et al., 2022; Bhattacharyya et al., 2014; Zhu et al., 2024). While *C. elegans* lacks certain inflammatory factors, its simple gene sequences make it suitable for initial screenings and pathway analyses (Kaletta and Hengartner, 2006).

Oxidative stress is a critical factor in UC pathogenesis, and our results show that baicalein significantly reduced ROS and MDA levels, both of which are markers of oxidative damage. Additionally, baicalein increased the activity of SOD, a key antioxidant enzyme, and reduced lipofuscin accumulation, suggesting that it not only protects against oxidative damage but also promotes healthier aging in *C. elegans* under DSS-induced stress conditions (Saul et al., 2021). However, the non-linear dose dependency of SOD activity—peaking at 50 μM but slightly declining at 100 μM despite oxidative damage reversal—suggests a multifactorial mechanism. At 25 μM, baicalein may primarily scavenge ROS directly through its phenolic hydroxyl groups, with minimal impact on SOD induction (Zuo et al., 2018). 50 μM baicalein likely activate transcriptional regulators like daf-16 or skn-1, upregulating SOD and other antioxidant enzymes (CAT) (Ayoub et al., 2024). At 100 μM, the plateauing or slight reduction in SOD activity—while maintaining significant efficacy over DSS controls—could reflect feedback inhibition (KEAP1-mediated Nrf2 degradation) or a shift toward non-enzymatic antioxidant mechanisms, which aligns with the hormetic responses typical of flavonoids (Wang et al., 2020). Critically, the absence of toxicity at 100 μM (supported by functional recovery of oxidative markers) underscores baicalein’s therapeutic window. Future studies should delineate the concentration-dependent crosstalk among these mechanisms to optimize dosing strategies that maximize antioxidative benefits without exceeding hormetic thresholds.

Baicalein’s therapeutic potential in UC is underpinned by its ability to modulate oxidative homeostasis and stress-responsive signaling networks. By targeting MAPK and PI3K/AKT pathways, baicalein restores intestinal redox balance, a critical factor in UC pathogenesis (Li J. et al., 2022; Tang et al., 2023). Our findings suggest its protective effects involves the IIS pathway, where it regulates DAF-16/FOXO transcription factors—key regulators of aging, immunity, and stress adaptation—downstream of DAF-2/IGF-1 (Accili and Arden, 2004). This FOXO homolog accumulates in the nucleus, where it directly binds to conserved DNA motifs (DBE/DIR elements) to coordinate stress response genes (sod-3, gst-4). Furthermore, in this study, 50 μM baicalein exhibited strong nuclear translocation ability. Mechanistically, local signaling networks, such as the Wnt/β-catenin pathway, may impose spatial constraints on DAF-16 activation by modulating its nuclear-cytoplasmic shuttling or transcriptional accessibility. Notably, high concentrations of baicalein (100 μM) could disrupt this regulatory balance, potentially through interference with Wnt/β-catenin-mediated intestinal homeostasis or feedback inhibition of IIS signaling (Mohanty and Suchiang, 2023). This hypothesis aligns with previous findings that gut-specific suppression of DAF-2 extends lifespan via enhanced DAF-16 nuclear localization, while excessive pharmacological intervention may override physiological signaling thresholds, leading to pathway desensitization or compensatory metabolic reprogramming. Further studies combining tissue-specific gene knockdown and pathway activity assays are warranted to dissect the interplay between IIS, Wnt signaling, and flavonoid-mediated lifespan modulation in intestinal epithelia (Zhang Y. P. et al., 2022). This mechanism aligns with the conserved role of FOXO homologs in mammals, which are essential for Treg cell differentiation and suppression of IFN-γ-driven inflammation in DSS colitis (Ohkura and Sakaguchi, 2010). Furthermore, the interplay between daf-16 and hif-1, another stress-responsive gene, underscores the evolutionary conservation of these pathways in maintaining barrier integrity under oxidative duress (Danese et al., 2022; Brown et al., 2020; Yin et al., 2022). Notably, HIF-1α (the mammalian counterpart of hif-1) cooperates with FOXO3 to enhance antioxidant defenses and epithelial repair, suggesting baicalein may exploit this synergy to mitigate UC progression.

Although baicalein modulates *daf-16*, it likely acts through additional complementary pathways, as the activity of *daf-16* is contingent on the phosphorylation of *akt-1*, a serine/threonine kinase downstream of PI3K. Phosphorylation of *akt-1*, which has been linked to chronic intestinal inflammation in IBD patients, suggests that baicalein’s protective effects might involve a more complex regulatory network, potentially modulating multiple signaling cascades (Zhang J. et al., 2022; Walldorf et al., 2021). Moreover PMK-1 may enhance DAF-16’s transcriptional activity by suppressing AKT activity, creating a feedback loop that amplifies stress resistance (Keshet et al., 2017).

Notably, sod-2 (mitochondrial SOD) and hsf-1 remained unaffected by baicalein in our model, despite their central roles in oxidative stress responses. It's likely that the expression of SOD-2 (mitochondrial superoxide dismutase) is typically regulated by redox-sensitive pathways (SIRT1), rather than Nrf2 or DAF-16 (Xiong et al., 2021). In addition, the competitive interaction between the HSF-1 and DAF-16 regulatory networks may prevent them from being simultaneously activated by the same stimulus, leading to the lack of induction of HSF-1 (Zhi et al., 2022). This complexity underscores that baicalein’s impact on intestinal inflammation and oxidative stress may not be fully explained by daf-2 and daf-16 alone.

This study adds to the growing body of evidence supporting the therapeutic potential of flavonoids, such as baicalein, in managing IBD. Flavonoids are known to enhance intestinal barrier function and modulate inflammatory responses through their effects on transcription factors and kinases such as PI3K/AKT and MAPK (Schwanke et al., 2013; Sahu et al., 2016). Importantly, while baicalein’s modulation of the DAF-2/IGF-1 and DAF-16/FOXO pathways plays a role, it is only one aspect of its broader mechanism of action. The involvement of the SKN-1/Nrf2 signaling cascade, activated by p38 MAPK in response to oxidative stress, adds another layer of complexity. This pathway is integral to innate immunity and longevity in *C. elegans*, suggesting that baicalein may enhance the organism’s ability to resist both oxidative and inflammatory damage through multiple molecular avenues (Dilberger et al., 2019; Hu et al., 2017; Tang and Pang, 2016; Liu F. et al., 2022). Thus, while the IIS and MAPK pathways are important targets, baicalein’s effects appear to be multifaceted, engaging a broader range of protective mechanisms.

**FIGURE 12:**
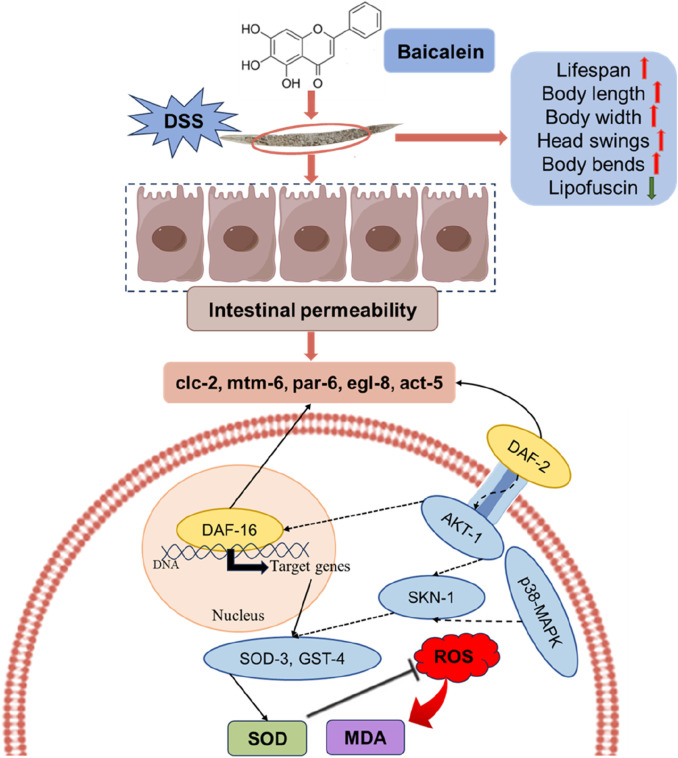
The proposed mechanisms by which baicalein mitigates the effects of DSS-induced damage in *C. elegans*. Upon DSS treatment, nematodes experience increased ROS and MDA levels, leading to oxidative stress and compromised intestinal permeability. Baicalein intervention appears to counteract these effects by enhancing several physiological parameters, including lifespan, body length, body width, head swings, and body bends, while decreasing lipofuscin accumulation, a marker of aging. In terms of oxidative damage, baicalein treatment enhances SOD activity, leading to reduced ROS levels, which subsequently lowers MDA formation, an indicator of lipid peroxidation. Additionally, baicalein improves intestinal permeability by modulating the expression of gene associated with barrier function. Mechanistically, the insulin/IGF-1 signaling pathway (IIS) and the MAPK pathway are involved in the regulation of baicalin oxidative stress and antioxidant capacity. This multi-pathway approach underscores baicalein’s potential as a therapeutic compound in enhancing health span and reducing oxidative stress under DSS-induced conditions.

While this study provides valuable insights into the protective mechanisms of baicalein, there are some limitations (Figure 12). We primarily focused on the effects of baicalein on intestinal barrier integrity and did not extensively examine downstream oxidative stress-related genes in epithelial cells. In mammals, DSS-induced colitis is characterized by chronic inflammation driven by adaptive immune responses (T-cell infiltration, cytokine cascades) and microbiome interactions (Zhang et al., 2025). In contrast, *C. elegans* lacks adaptive immunity and a microbiota, rendering DSS effects primarily acute, involving innate immune pathways (e.g., IIS, p38 MAPK), oxidative stress, and direct epithelial damage. And acute DSS exposure in worms mirrors early epithelial injury phases, whereas mammalian models replicate chronic inflammation with crypt hyperplasia and fibrosis. These differences highlight the utility of *C. elegans* for dissecting evolutionarily conserved pathways of epithelial stress and innate immunity, albeit with limited translatability to chronic or adaptive immune-mediated inflammation. To address species-specific limitations and strengthen pathological relevance, we propose the following refinements in subsequent studies: Dual-damage challenge: Co-exposure to DSS and pathogenic bacteria (e.g., OP50) to activate *C. elegans* p38 MAPK signaling and quantify antimicrobial peptide expression (abf-2, nlp-29) via qRT-PCR. Tissue-specific immune monitoring: Utilize transgenic reporters (e.g., irg-1pGFP) to dynamically assess intestinal immune activation *in vivo*. Future studies should leverage complementary models to bridge acute mechanistic insights with complex pathophysiology. It should aim to explore these pathways in greater detail, as well as validate the findings in mammalian models to further substantiate baicalein’s therapeutic potential in UC.

## 5 Conclusion

The findings of this study demonstrate that baicalein confers protective effects against ulcerative colitis (UC) in a DSS-induced *C. elegans* model, primarily through enhancement of intestinal barrier integrity and mitigation of oxidative stress. Mechanistically, these benefits are associated with modulation of the p38/MAPK and insulin/IGF-1 signaling (IIS) pathways, underscoring baicalein’s dual role in attenuating inflammatory and oxidative damage. Furthermore, this work validates the utility of the *C. elegans* DSS model as a robust preclinical tool for rapid screening of UC therapeutics. The evidence presented here positions baicalein as a promising candidate for further investigation in higher-order models, with emphasis on its translational potential in targeting pathway-specific dysregulation characteristic of UC pathogenesis.

## Data Availability

The original contributions presented in the study are included in the article/Supplementary Material, further inquiries can be directed to the corresponding author.
