# Supplementary material for: Baicalein from Scutellaria baicalensis mitigates oxidative stress through the IIS pathway in a C. elegans model of ulcerative colitis
Source: Front Pharmacol. 2025 Jun 26;16:1592244. doi: 10.3389/fphar.2025.1592244 (PMC12241015; doi:10.3389/fphar.2025.1592244)
Supplement: Supplementary file 1 [file Table1.docx]

Table 1. Sequences of the oligonucleotide primers for qRT-PCR

| Gene | Primer sequence |
| --- | --- |
| clc-2 | Forward: 5’-CAAGATAACCATAATCCGCTTTGAACC-3’ |
|  | Reverse: 5’-GCAATACTCGTCCTCACCATAATCG-3’ |
| act-5 | Forward: 5’-CCTCTTCCAGCCTGCCTTCATC-3’ |
|  | Reverse: 5’-AGAACAGTGTTGGCGTAGAGATCC-3’ |
| mtm-6 | Forward: 5’-AACGCTTGAGGCTTCTGGATGG-3’ |
|  | Reverse: 5’-TCCGAACAATGAACGACACATGATG-3’ |
| par-6 | Forward: 5’-CACTGATATGATGGTCGCCAATGC-3’ |
|  | Reverse: 5’-TGTTCCTTGTTGTGACGGTCCTC-3’ |
| egl-8 | Forward: 5’-GATTTCCTCCGAAGACCCGACAG-3’ |
|  | Reverse: 5’-CCGAGATGACACGAACACTACAATG-3’ |
| skn-1 | Forward: 5’-GGTAGCCGACGACGAAGAAGAG-3’ |
|  | Reverse: 5’-TGGATTGAGGTGTTGGACGATGG-3’ |
| akt-1 | Forward: 5’-TGGTAAGATGCCTTCAGTGGACAAC-3’ |
|  | Reverse: 5’-GAATCCAACGCTGACGAACTTCTG-3’ |
| gst-4 | Forward: 5’-TCCGTCAATTCACTTCTTCCG-3’ |
|  | Reverse: 5’-CACCATGAGTCCAATGATTGCA-3’ |
| sod-3 | Forward: 5’-CGAGCTCGAACCTGTAATCAGCCATG-3’ |
|  | Reverse: 5’-GGGGTACCGCTGATATTCTTCCAGTTG-3’ |
| sod-2 | Forward: 5'-TCGCTGTTCAAGGATCAGGATGG-3’ |
|  | Reverse: 5'- CAAGTCCAGTTGTTGCCTCAAGTG-3’ |
| daf-2 | Forward: 5’-GTTGATAATGCTGCCGAG-3’ |
|  | Reverse: 5’-ATCCCGGTCCGATTTCTT-3’ |
| daf-16 | Forward: 5’-ATCGTGTGCTCAGAATCC-3’ |
|  | Reverse: 5’-ATGAATAGCTGCCCTCC-3’ |
| hif-1 | Forward: 5’-AGCTGCTCGGCCTGTGC-3’ |
|  | Reverse: 5’-TTGCTGTTGCTGTCCTTGCC-3’ |
| hsf-1 | Forward: 5’-TCGGCAAGAACTGTGGAGGAC-3’ |
|  | Reverse: 5’-CGCATCGCACGCATCTCTG-3’ |
| sek-1 | Forward: 5'-CGAGTCCGAAGAGATTGAGATTGC-3’ |
|  | Reverse: 5'- CCCGCTCTGCCTGTGCTG-3’ |
| nsy-1 | Forward: 5'-GTATTGGTGTTGGCGGAGGTTC-3’ |
|  | Reverse: 5'- ACTGGCTGACGGCGTTGAC-3’ |
| pmk-1 | Forward: 5'-TGCTGACGAAGAGGAAGATGAGG-3’ |
|  | Reverse: 5'- AACTGCCTTGGGATTGTAAGATGTC-3’ |
| act-1 | Forward:5’-TCGGTATGGGACAGAAGGAC-3’ |
|  | Reverse: 5’-CATCCCAGTTGGTGACGATA-3’ |

Note: The concentration of all primers is 10 μM.

Table 2. Effect of different baicalein concentrations on longevity of nematodes treated with 5% DSS (day)

| Group | Longevity of nematodes (day) | p-value |
| --- | --- | --- |
| Control | 10.000±0.764 | - |
| 5%DSS | 7.270±0.565 | 0.005^*^ |
| Baicalein-L (25 μM) | 9.027±0.714 | 0.106 |
| Baicalein-M (50 μM) | 9.784±0.785 | 0.009^##^ |
| Baicalein-H (100 μM) | 8.351±0.593 | 0.224 |

Note: Statistical significance is denoted by '*' for comparisons with the control group and '#' for comparisons with the 5% DSS treatment group, with *p < 0.05, ***p < 0.001, #p < 0.05 and ##p < 0.01 indicating significance levels.

Table 3 Effects of different baicalein concentrations on body length and width of 5% DSS treated nematodes

| Group | Body length (μm) | | Body width (μm) | |
| --- | --- | --- | --- | --- |
|  | Mean±SD | p-value | Mean±SD | p-value |
| Control | 657.85±2.46 | - | 47.01±0.37 | - |
| 5%DSS | 637.48±3.44 | 0.016^*^ | 44.14±0.26 | 0.001^***^ |
| Baicalein-L (25 μM) | 653.99±2.25 | 0.102 | 45.69±0.28 | 0.057 |
| Baicalein-M (50 μM) | 665.79±2.22 | 0.016^#^ | 46.19±0.31 | 0.012^#^ |
| Baicalein-H (100 μM) | 668.90±2.46 | 0.001^###^ | 46.30±0.31 | 0.008^##^ |

Note: Statistical significance is denoted by '*' for comparisons with the control group and '#' for comparisons with the 5% DSS treatment group, with *p < 0.05, ***p < 0.001, #p < 0.05, ##p < 0.01, and ###p < 0.001 indicating significance levels.
